# Randomised controlled trials for evaluating the prescribing impact of information meetings led by pharmacists and of new information formats, in General Practice in Italy

**DOI:** 10.1186/1472-6963-7-158

**Published:** 2007-09-28

**Authors:** Nicola Magrini, Giulio Formoso, Anna Maria Marata, Oreste Capelli, Emilio Maestri, Claudio Voci, Francesco Nonino, Massimo Brunetti, Barbara Paltrinieri, Susanna Maltoni, Lucia Magnano, Maria Isabella Bonacini, Lisa Daya, Nilla Viani

**Affiliations:** 1 Centre for the Evaluation of the Effectiveness of Health Care, Azienda USL; Viale Muratori 201; 43100 Modena, Italy

## Abstract

**Background:**

Suboptimal translation of valid and relevant information in clinical practice is a problem for all health systems. Lack of information independent from commercial influences, limited efforts to actively implement evidence-based information and its limited comprehensibility are important determinants of this gap and may influence an excessive variability in physicians' prescriptions. This is quite noticeable in Italy, where the philosophy and methods of Evidence-Based Medicine still enjoy limited diffusion among practitioners. Academic detailing and pharmacist outreach visits are interventions of proven efficacy to make independent and evidence-based information available to physicians; this approach and its feasibility have not yet been tested on a large scale and, moreover, they have never been formally tested in Italy.

**Methods/Design:**

Two RCTs are planned:

1) a two-arm cluster RCT, carried out in Emilia-Romagna and Friuli Venezia Giulia, will evaluate the effectiveness of small group meetings, randomising about 150 Primary Care Groups (corresponding to about 2000 GPs) to pharmacist outreach visits on two different topics. Physicians' prescriptions (expressed as DDD per 1000 inhabitants/day), knowledge and attitudes (evaluated through the answers to a specific questionnaire) will be compared for target drugs in the two groups (receiving/not receiving each topic).

2) A three-arm RCT, carried out in Sardinia, will evaluate both the effectiveness of one-to-one meetings (one pharmacist visiting one physician per time) and of a 'new' information format (compared to information already available) on changing physicians' prescription of specific drugs. About 900 single GPs will be randomised into three groups: physicians receiving a visit supported by "traditional" information material, those receiving a visit with "new" information material on the same topic and those not receiving any visit/material.

**Discussion:**

The two proposed RCTs aim to evaluate the organisational feasibility and barriers to the implementation of independent information programs led by NHS pharmacists. The objective to assess a 10 or 15% decreases in the prescription of the targeted drugs is quite ambitious in such 'natural' settings, which will be minimally altered by the interventions themselves; this in spite of the quite large sample sizes used comparing to other studies of these kind. Complex interventions like these are not easy to evaluate, given the many different variables into play. Anyway, the pragmatic nature of the two RCTs appears to be also one of their major strengths, helping to provide a deeper insight on what is possible to achieve – in terms of independent information – in a National Health System, with special reference to Italy.

**Trial registration:**

ISRCTN05866587 (cluster RCT) and ISRCTN28525676 (single GPs RCT)

## Background

Information that doctors receive on benefits and risks of currently used drug treatments generally comes, either directly or indirectly, from the pharmaceutical industry [1-3]. It may thus be unsystematic and biased [4], and may be a component of the (often observed) excessive variability in physicians' prescriptions [5,6], uneasy to explain if one merely looks at evidence-based findings in medical literature.

In recent years the Italian Ministry of Health has made efforts in expanding physicians' access to independent and evidence-based information, like freely distributing the Italian translation of Clinical Evidence and enhancing the quality of the already freely distributed NHS Drug Information Bulletin. Nonetheless, sub-optimal diffusion of independent scientific information is apparent, as well as its comprehensibility to physicians who do not generally have epidemiology and statistics in their medical school background [7-11]. Improving access to, as well as comprehensibility of, evidence-based information remain important goals for Health Authorities, both at national and local levels.

However, simple diffusion of information – even if "evidence-based" – does not seem to affect prescribing behaviour, whereas active interventions like educational outreach visits to doctors (either one to one or small groups meetings), often employing pharmacists, seem more effective especially when clinical information is supplemented by prescribing data, according to an "*audit & feedback*" method [12-16].

Hence, in Italy there is room for improving independent information initiatives, promoting an active outreach system and a different role for NHS pharmacists, the latter being too often confined in administrative/auditing roles. There is also room for improving comprehensibility of medical information, which is infrequently targeted to "average" doctors and hardly ever offered in a clear, direct and appealing fashion, looking at the context of clinical practice [17].

The available evidence on the effectiveness of outreach visits comes from the English speaking world (United Kingdom, Canada, Australia, United States), whereas no studies have formally evaluated their effectiveness, feasibility and perceived usefulness in Italy [12-14]. For this reason, two pragmatic randomised controlled trials (RCTs) are proposed to carry out this complex evaluation in different settings: the first one, carried out in Emilia-Romagna and Friuli Venezia Giulia, will be a cluster RCT evaluating whether information meetings led by pharmacists with small groups of physicians change their prescribing behaviour, attitude and knowledge (being a cluster design the most appropriate to address the issue of contamination between groups); whereas the second one, carried out in Sardinia, will be a RCT evaluating one to one visits and the added value of a new format in improving doctors' knowledge, attitudes and prescribing practice [17].

Two more reasons to carry out these studies are that they would represent an unprecedented effort to accomplish such evaluation on quite a large scale, considering that three Italian regions would participate (Emilia Romagna with about 1500 physicians, assisting a population of about 2 million inhabitants; Friuli Venezia Giulia with about 400 physicians, assisting a population of about 400,000; and Sardinia with about 900 physicians, assisting a population of about 1 million) and that these interventions would be carried out in a 'natural' organizational setting, which would be minimally altered by the interventions themselves.

### Objectives

Overall, the two studies will specifically evaluate:

• whether information meetings with small groups of physicians (in Emilia-Romagna and Friuli Venezia Giulia) or with single physicians (in Sardinia), led by pharmacists and organised by Local Health Authorities within a large scale independent information program involving all local GPs, is organisationally feasible (and how barriers can be reduced)

• whether such programs can be effective in changing physicians' prescribing behaviour

• whether the provided information can also modify knowledge and attitudes of physicians on benefits and risks of the drugs under scrutiny (in Emilia-Romagna and Friuli Venezia Giulia)

• whether the format of information can affect the outcomes, specifically: whether an "enriched" format targeted at the average GP can improve knowledge, attitudes and prescribing behaviour more than a "traditional" format (in Sardinia)

• whether an active role of a GP (delegate by the PCG) in information meetings is feasible and useful (in Emilia-Romagna and Friuli Venezia Giulia)

## Methods/Design

### Study population

#### Emilia-Romagna and Friuli Venezia Giulia study (cluster RCT)

Primary Care Groups (PCGs) will be the unit of randomisation (cluster). PCGs are defined as small groups, ranging from about 10 to 20 general practitioners (GPs) and assisting about 8,000 to 25,000 people in a defined area. A general rule is to include PCGs with less than or equal to 20 GPs. Exceptions may be accepted (considering the pragmatic nature of the study) but should be adequately justified by Local Health Authorities recruiting them. In any case, the proposal to split groups with more than 20 subjects will be made.

Local Health Authorities, which are in charge of the local organization of the study, should actively promote the information meetings by making them part of compulsory education and/or providing CME credits.

#### Sardinia study (randomising single GPs)

Single GPs will be the unit of randomisation. All GPs are eligible, provided that their Local Health Authority has an organised system for tracking their prescriptions made within the NHS till the 5^th ^ATC level, in order to evaluate these prescriptions and to provide doctors with feedback. As in the first study, Local Health Authorities should actively promote the outreach visits among single GPs.

### Intervention/Exposure

#### Emilia-Romagna and Friuli Venezia Giulia study (cluster RCT)

A cluster RCT will be performed. PCGs will participate to two rounds of small group information meetings where a trained pharmacist, possibly supported by a GP (a "referent", delegate by the PCG), will provide information, discussing the contents of a bulletin specifically developed about the main studies/literature available on benefits and risks of drugs for preventing and/or curing a specific disease (enriched format) [17].

**Figure 1:**
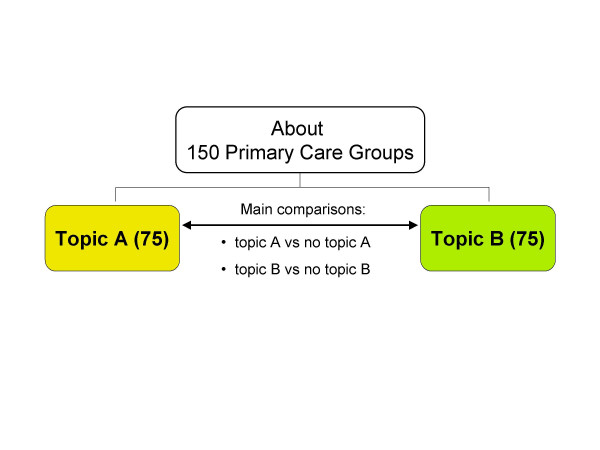
Design of the cluster study (Emilia-Romagna and Friuli Venezia Giulia): effectiveness of information meetings.

Within each round of visits (taking place in spring and autumn of 2007, respectively), PCGs will be randomised in two groups: those receiving information about a specific topic and those not receiving it (Fig. 1). To improve acceptability to Local Health Authorities and sample efficiency, PCGs not receiving information about one specific topic (e.g. topic A) will receive another topic (e.g. topic B). Two evaluations will then be carried out: for topic A related outcomes, recipients vs non recipients of topic A will be compared; conversely, for topic B related outcomes, the comparison will be between recipients vs non recipients of topic B.

Considering two rounds of information meetings, four topics will be needed (two topics each round) and four interventions will be tested overall. While these are different specific comparisons, the same general hypothesis will be tested: whether information meetings led by pharmacists are effective in changing physicians' behaviour (comparing to no visit on the same topic). The reasons why four comparisons are planned are to better evaluate the feasibility of this kind of intervention (which could be eventually proposed in the Italian NHS on a routine basis) and to have better insight into its effectiveness, since this may depend on the kind of topics and on the extent of information presented.

The first couple of topics chosen (for the spring 2007 round) are: 'drugs for the treatment of benign prostatic hyperplasia', and 'drugs for the primary prevention of osteoporotic fractures in elderly people'. The second couple of topics will be chosen about two-three months before the autumn 2007 round and this choice will depend, as well as for the first two topics, on relevance for clinical practice, limited availability and diffusion of information independent from drug industry, possibility to synthesize clear and evidence-based messages on benefits and risks of therapies and possibility to affect drug prescriptions significantly.

Stratified randomisation will be carried out within each Local Health Authority (LHA), using the number of assisted population per PCG (under or over the mean for that LHA) as stratifying factor. Pharmacists will be randomised along with the PCGs since the meeting scheme (which pharmacist meeting which PCG) will be defined before randomisation: this in order to avoid a 'topic-based' self-selection of visiting pharmacists, which may introduce a bias (doctors receiving intervention A could meet more or less motivated pharmacists, than those receiving intervention B).

About one pharmacist every 8–10 PCGs will get ad hoc training for the study, getting:

• one four-day intensive course (36 hours) on EBM methodology

• one three-day course (about 20–22 hours) for each of the four selected topics, discussing about the benefits and risks of drugs used in the correspondent clinical area and their relevance to clinical practice, looking at the available studies and at their internal/external validity.

Each PCG will nominate a "referent" GP to support pharmacists during the information meetings. Referents will get a half-day training module each round on the topic their group has been randomised to.

#### Sardinia study (randomising single GPs)

**Figure 2:**
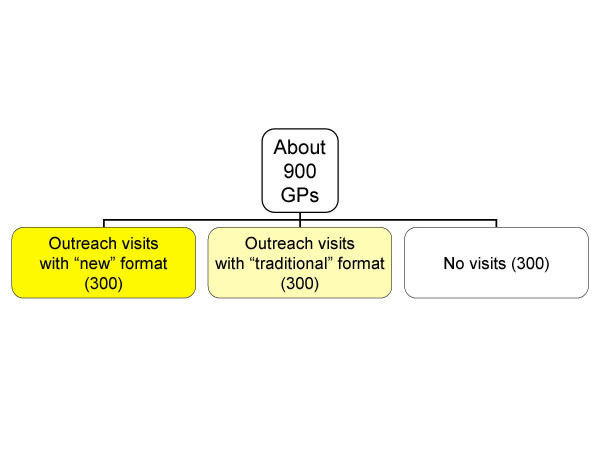
Design of the single doctor study (Sardinia): effectiveness of outreach visits and of two different formats.

A three-arm RCT is planned: single GPs will be randomised so that they may receive: an outreach visit from a trained pharmacist, supported by information material in a 'traditional' format as background material; the same kind of visit supported by an 'enriched' format (the type of bulletin used in the cluster study) [17]; or no visit at all (Fig. 2). Both the effectiveness of the outreach visit and of the information format will be evaluated (see outcomes).

Two rounds of visits are planned in this study too, each focusing on one topic. Therefore, only two topics will be addressed overall (comparing to four used for the other study). The first topic chosen is 'drugs for the treatment of benign prostatic hyperplasia'.

The 'traditional' material for this round has been selected considering its availability and diffusion and its recognised dependability. Two articles on that topic available in other drug bulletins have been eventually translated into Italian to be used for this scope: they appeared on the issue n. 252 of La Revue Prescrire ^® ^and on the issue n. 58 of Therapeutics Letter ^® ^About one pharmacist every 80 GPs will receive the same training as the pharmacists in the other study.

A graphical display of study design is shown in fig. 1 (cluster RCT) and fig. 2.

### Outcomes

#### Main outcome

difference (%) in NHS prescription of drugs under scrutiny (expressed as DDD per thousand inhabitants/day), comparing those who have/do not have received the specific information. In case of relevant baseline unbalances, statistical adjustment will be sought

#### Secondary outcomes

• difference in the % of patients who were prescribed the specific drug(s)

• difference in the % of patients who were prescribed the specific drug(s) for the first time (in the previous 12 months)

• difference in expenditure for the specific drug (per 1000 patients/day)

• difference in NHS prescription of drugs under scrutiny (expressed as DDD per thousand inhabitants/day), comparing those who have received the "traditional" vs "enriched" format, and any information vs no information (Sardinia study only)

• differences in the main and secondary outcomes in each of the regions involved (cluster study only);

• differences in the main and secondary outcomes according to the n. of assisted population in the related PCG (cluster study only)

• differences in the main and secondary outcomes according to the terziles of physicians age

• adjusted difference in prescribed DDD per 1000 patients/day according to a statistical model, considering as possible covariates: overall prescription in DDD per 1000 patients day at baseline; n. of physicians in the specific PCGs; n. of assisted population; region (Emilia-Romagna or Friuli Venezia Giulia); geographical location (mountain, hill, plain, urban centre – according to definitions given by the Italian Statistics Institute); age distribution of assisted population (in quartiles); % females in the assisted population; physician age; total physician drug expenditure (excluding drugs under scrutiny); % assisted population with polyprescription (≥ three drugs of different classes); n. new prescriptions (in the last 12 months); month of evaluation; participation to the information meetings; % exact answers to the questionnaire testing knowledge (see additional file 1);

• difference in the variability (expressed as standard deviations) of prescription of drugs under scrutiny within PCGs (cluster study only)

• difference in knowledge (measured through the n. of correct answers to a specific anonymous questionnaire, see additional file)

• difference in attitudes (measured through the answers to a specific questionnaire, see additional file)

Drugs whose NHS prescription will be considered for the first two topic chosen are specified here below:

1. for benign prostatic hyperplasia, the main indicators will be: difference in the prescription of 5 alpha-reductase inhibitors (finasteride and dutasteride, for which a reduction is expected); and difference in the ratio between the prescription of alpha blockers terazosin plus tamsulosin (already off patent drugs) versus other drugs of the same classes available in Italy (alfuzosin plus doxazosin – an increase of this ratio is expected). Secondary indicators will be: difference in the prescription of each of the alpha blockers listed above (a reduction is expected); difference of concomitant prescription of 5 alpha-reductase inhibitors and alpha blockers; and overall difference in expenditure for either 5 alpha-reductase inhibitors or alpha blockers (a reduction is expected for both of these indicators).

2. for the primary prevention of osteoporotic fractures, the main indicators will be: difference in the prescription of either alendronic acid or of sodium risedronate (a reduced increase is expected for both of these indicators, especially for the latter – for which the generic form is still unavailable). Secondary indicators will be: difference in the prescription of raloxifene and strontium ranelate (a reduction is expected for both of these indicators); and difference in the ratio between the prescription of alendronic acid (already off patent) versus the other two oral bisphosphonates available in Italy (sodium risedronate and ibandronic acid – an increase of this ratio is expected)

Physicians' expectations, perceived barriers and the added value about the proposed intervention will be eventually evaluated through focus groups with a sample of participating doctors for each region (at the end of the spring and the autumn rounds).

### Economic analysis

If information meetings with small groups of physicians (*Emilia-Romagna and Friuli Venezia Giulia study*) or with single physicians (*Sardinia study*) will be more effective than no meetings, we will conduct economic evaluations for each study to evaluate the opportunity cost of our interventions.

We will adopt the perspective of the National Health Service, not considering indirect costs [18]. As we expect no health consequences of the changes in prescribing patterns, we will conduct a cost-minimisation analysis evaluating whether the savings on drug-costs are greater than the costs of the intervention.

*In the Emilia-Romagna and Friuli Venezia Giulia study we will consider *the cost of the evidence based bulletins specifically developed. In the *Sardinia study we will consider both *the cost of the evidence based bulletins specifically developed and the cost of the translation of the articles used as "traditional" information material.

All costs will be reported in monetary as well as natural units. An overview of all included costs and data sources is presented in Table 1. As all costs occur within the same year, we will not apply discounting.

**Table 1:** Input variables in the economic analysis

**Variable**	**Data-source (natural units)**	**Source for monetary units**
Cost for development of evidence based material and translation of drug bulletin	Time spent	Salary payments
Training of outreach pharmacists	Time spent	Salary payments
Training of 'referents' (physicians indicated by each PGC)	Time spent	Salary payments
Printed materials	Invoices	Invoices
Travels costs	Estimate of distance to practices	Travel invoices
Cost of pharmacists doing outreach and making appointments	Record of number of visits and days spent on visits	Salary payments
Cost of other administrative tasks	Estimated time expenditure	Salary payments
Cost of physician time	Record of length of outreach visit and number of physicians	Salary payments
Development of software	Invoices, estimates of time spent	Invoices, salary payments
Drug expenditure	Medical records of prescribing	Italian Agency for Drug 2006

We will perform a sensitivity analysis, considering how variations of different parameters can affect results. Specifically, on the cost side we will consider different values in both quantities and prices of the consumed resources. On the effectiveness side, we will consider the boundaries of the confidence intervals. Moreover, we will perform best case/worst case analysis considering incremental values for cost and effectiveness (lower costs and better effectiveness vs higher costs and lower effectiveness).

### Follow-up

The follow-up time will be six months after each intervention. A further evaluation will be carried out after 12 months to assess persistence

### Information retrieval

Monthly prescription data will be retrieved from provincial drug prescription databases. Although impossible to eliminate completely, information bias due to database accuracy should not affect our evaluation significantly, since limited error rates are expected.

In the cluster RCT, knowledge and attitudes of physicians will be evaluated through separate questionnaires distributed during the information meetings. Each set of questions will be topic dependent, therefore questionnaires will be developed for each topic. The additional file shows the questionnaires related to the first two topics.

The knowledge questionnaire will be 'nominal' and filling up it will be necessary for receiving CME credits, while the attitude questionnaire will be anonymous so that responders can express their views freely.

### Monitoring of the study

After each meeting, pharmacists will report basic information (n. of participating physicians, duration, critical questions, active participation of 'referents', active participation to discussion, n. returned questionnaires, etc) on an online database. The coordinating centre (CeVEAS) will check that meetings are carried out according to randomisation by monitoring the database, showing up to about 10% of the meetings at random (for the cluster study) and contacting physicians by phone (for the Sardinia study)

### Sample size estimates

#### Emilia-Romagna and Friuli Venezia Giulia study (cluster RCT)

From a sample of 40 clusters (about 600 physicians) and using a dedicated software (Cluster Randomisation Sample Size Calculator version 1.0.2, Health Services Research Unit, Aberdeen University) we estimated the intracluster correlation coefficients (ICC) and the sample size that would be required to see a difference of 10% in the prescription of a number of possible drugs (15% for bisphosphonates), assuming an average cluster (PCG) size of 15 (Table 2).

**Table 2:** Estimates of sample sizes to detect a prescribing difference of 10% (with two-tailed test)

	ATC code	Estimate of ICC	Sample size (NCP)
macrolides	J01FA	0.024	68
fluoroquinolones	J01MA	0.030	68
antidepressants	n06ab	0.058	88
nitroderivatives	C01da02, 05,08,14	0.09	96
bifosphosphonates **(15% diff)**	M05ba	0.062	106
proton pump inhibitors	A02BC	0.087	108
phosphomicine	J01xx01	0.014	108
Alfa blockers (for IPB)	g04ca + g04bx49	0.075	116
doxazosin (for IPB)	c02ca04	0.038	128
long acting β2 broncodilators + corticosteroids	R03AK06 e R03AK07	0.092	148
Finasteride +dutasteride	g04cb	0.08	154
opioids	N02A	0.061	164
clarithromicine	J01fa09	0.02	172
esomeprazole	A02bc05	0.023	204
long acting β2 broncodilators	R03AC12 e R03AC13	0.033	208
alendronate	M05ba04	0.08	218
finasteride	g04cb01	0.1	220
salmeterol + corticosteroids	R03ak06	0.102	234
bifosphosphonates	M05ba	0.062	238
azitromicine	J01fa10	0.068	266
risedronate	M05ba07	0	374
norfloxacine	J01ma06	0.055	386
fentanyl	N02ab03	0.043	428
salmeterol	R03ac12	0.053	698
morphine	N02aa01	0.05	2224
buprenorphine	N02ae01	0	Nc
bicalutamide	l02bb03	0	Nc
escitalopram	N06ab10	0	1434
levocetirizine	R06ae09	0.032	>600
antiandrogens	L02bb01+ L02bb03	0	nc

Since we expect to recruit about 150 PCGs we choose for the first round of information meetings the two topics previously described ('drugs for the treatment of benign prostatic hyperplasia', and 'drugs for the primary prevention of osteoporotic fractures in elderly people') because of the clinical relevance of the information to be presented.

#### Sardinia study (randomising single GPs)

We expect to recruit about 900 General Practitioners. Such a sample would exceed the number needed to assess the same objectives described for the other study.

### Statistical analysis

The statistical analysis will be performed according to the intention-to-treat principle, analysing physicians' prescriptions according to the treatment group to which they were randomly allocated (as single GPs or as members of PCGs), and independently from their participation to the outreach visits. A 'modified intention to treat' will be considered in case some physician should not be actually offered the randomised intervention (for example, if pharmacists should become unavailable in a given area for organisational reasons or if health policy issues should arise, e.g. lack of agreement with medical syndicates, etc). Secondly, a by treatment analysis will also be performed considering only physicians who actually participated to the visits.

T-test (for continuing variables), chi-square test (for discrete variables) and multiple linear regression will be used, the latter to investigate how specific variables (like baseline prescriptions, region, size of PCG, size of assisted population, age and sex of GP, month of follow-up, etc) can affect outcomes. Analysis of covariance (ANCOVA) will be used for evaluating primary outcomes in case relevant baseline imbalances (for example in the prescription of targeted drugs) will make statistical adjustment necessary.

### Organizational characteristics

CeVEAS will coordinate and monitor all the aspects of the study and will be in close connection with the Health Authorities in charge of the local organization. CeVEAS will also provide the necessary training to the pharmacists involved, and will carry out the final evaluation of the study. Regional Health Authorities of Emilia-Romagna, Friuli Venezia Giulia and Sardinia will help select the Local Health Authorities where the study will be carried out, and will participate to the local organization of the project. Local Health Authorities (LHAs) will ensure the local organization, selecting "outreach" pharmacists, promoting their training, and promoting and scheduling the outreach visits. LHAs will also ensure the retrieval of drug prescribing data for the entire study period. Regional and Local Health Authorities do not need previous experience in setting outreach visits programs, the only requirement being the availability of reliable drug prescription databases for retrieval of GPs' prescribing data.

### Steering Committee

A Steering Committee (SC) was nominated in September 2006 to approve the final version of the protocol and to address ethical matters. Specifically, the SC will supervise study organisation, conduct and analysis of results, and will be made of 16 components: a chairman; three representatives of the coordinating centre; one representative of the Regional Health Authority, two representatives of GPs and one representative of pharmacists for each of the three participating regions. Specifically the SC will:

• verify that all the Health Authorities involved adhere to the minimum requirements indicated in the study protocol, in keeping with the pragmatic nature of the study

• verify that data have been adequately checked for quality

• supervise the production of reports to inform the participating Health Authorities about the progression of the study

• monitor the scientific literature to seek for possibile new relevant information on the discussed topics

• evaluate possible amendments to the protocol

## Discussion

The two proposed RCTs will let us better understand the potential usefulness of NHS pharmacists assuming a more active role in transferring evidence-based information to physicians. Besides data on prescribing impact, the studies will provide qualitative insight on the feasibility of such information programs, with special attention to how different organizational settings in different regional areas may lead to better or worse implementation. As already said, these studies represent an unprecedented effort to accomplish such evaluations on quite a large scale and in a 'natural' organizational setting, which would be minimally altered by the interventions themselves. Moreover, the two RCTs will test two different strategies of information delivery: using small groups or one to one meetings. While these strategies will not be directly compared (being not employed in the same study but in two different ones), their feasibility and impact will be critically evaluated, also in light of other experiences described in the scientific literature and of methods used by the pharmaceutical industry to bring information to doctors on a large scale.

Last but not the least, the role of the format of medical information will be investigated: this could be a critical part in the implementation of information programs and its importance may be often underestimated, considering that medical schools generally do not offer the necessary tools to unscramble data published in the scientific literature; that average doctors do not have enough time to select and read what may add relevant information to their knowledge; and that, eventually, the uptake of this information may be facilitated by offering it in a clear, direct and appealing fashion, looking at the context of clinical practice [17].

As usually happens, the evaluation of complex interventions is intricate given the many different variables into play: it is the other side of the pragmatic nature of these RCTs, which is also, and undoubtedly, one of their strengths. Moreover, the ambitious quantitative hypothesis to be tested (10 or 15% difference in doctors' prescriptions) will not be easily demonstrated, in spite of the involvement of about 2000 physicians in the cluster RCT and about 900 in the single doctors RCT: an even larger sample size would have been necessary to see smaller but still relevant prescribing differences.

### Feasibility

The Centre for the Evaluation of the Effectiveness of Health Care (CeVEAS), a NHS Centre dedicated to the production and implementation of independent information, has already been implementing outreach visit programs in selected areas of Emilia-Romagna starting from 2001, using "Information Packages on Drugs" ^® ^(drug information bulletins, registered in the International Society of Drug Bulletins since 2006, developed to facilitate knowledge transfer). However, the large scale feasibility of such information program has not been formally tested, neither a formal impact assessment on physicians' knowledge, attitudes and prescribing practice has been carried out.

### Ethical aspects

Between November 2006 and March 2007, the protocol was sent to the Local Ethics Committees (LEC) of the Health Authorities involved. Most of the LEC have already approved the protocol, some specifying that it was unnecessary to analyse it formally since no ethical problems arise in carrying out a randomisation differentiating the kind of information actively discussed during the outreach visits. In any case, the same information will be available to everybody outside the study meetings. At the end of each follow-up period, the information discussed during outreach visits will be proactively sent to each physician unexposed to that specific information during the outreach visits.

## Competing interests

Since 2001, CeVEAS has been implementing pharmacist outreach visit programs in a few Health Districts of Emilia-Romagna, receiving support and NHS funding from the corresponding Local Health Authorities, and is interested in devising new tools for facilitating knowledge transfer.

## Authors' contributions

NM proposed the studies, provided substantial contribution to their design, provided feedback for the manuscript and gave final approval of the version to be published. He is the study guarantor and is supervising the choice and development of the information and educational interventions (Information Packages and related training).

GF proposed and designed the studies and wrote the study protocol, giving final approval of the version to be published. He is supervising the studies and participating in the choice and development of the information and educational interventions (Information Packages and related training).

AM participated in designing the studies and gave final approval of the version to be published. She is supervising the studies and the choice and development of the information and educational interventions (Information Packages and related training).

OC participated in designing the studies and gave final approval of the version to be published. He is coordinating the cluster study participants in Emilia-Romagna and participating in the choice of the information and educational interventions (Information Packages and related training). He gave substantial contribution to the development of the Information Package on "Drugs for benign prostatic hyperplasia".

EM participated in designing the studies and gave final approval of the version to be published. He is participating in the choice of the information and educational interventions (Information Packages and related training). He gave substantial contribution to the development of the Information Package on "Drugs for primary prevention of osteoporotic fractures".

CV participated in designing the statistical analyses, supervised the randomisation processes and gave final approval of the version to be published. He is coordinating the data flow and carrying out the statistical analyses.

FN read and gave final approval of the version to be published. He is coordinating the cluster study participants in Friuli Venezia Giulia and participating in the development of the information and educational interventions (Information Packages and related training).

MB proposed and drafted the section addressing the economic analyses. He read and gave final approval of the version to be published. He is coordinating the study participants in Sardinia.

BP read and gave final approval of the version to be published. She is participating in the development of the information and educational interventions (Information Packages and related training) and coordinating data monitoring.

SM read and gave final approval of the version to be published. She is participating in the development of the information and educational interventions (Information Packages and related training).

LM read and gave final approval of the version to be published. She is participating in the development of the information and educational interventions (Information Packages and related training).

MIB read and gave final approval of the version to be published. She is participating in the development of the information and educational interventions (Information Packages and related training).

LD read and gave final approval of the version to be published. She is participating in the development of the information and educational interventions (Information Packages).

NV read and gave final approval of the version to be published. She is participating in the development of the information and educational interventions (selection and translation of the international drug bulletins used as comparators).

## Pre-publication history

The pre-publication history for this paper can be accessed here:



## Supplementary Material

Additional file 1 example of questionnaire on knowledge and attitudes. example of questionnaires testing knowledge and attitudes of doctors receiving information on Benign Prostatic Hyperplasia. Click here for file
