# Supplementary material for: Randomised controlled trials for evaluating the prescribing impact of information meetings led by pharmacists and of new information formats, in General Practice in Italy
Source: BMC Health Serv Res. 2007 Sep 28;7:158. doi: 10.1186/1472-6963-7-158 (PMC2100051; doi:10.1186/1472-6963-7-158)
Supplement: Additional file 1 — example of questionnaire on knowledge and attitudes. example of questionnaires testing knowledge and attitudes of doctors receiving information on Benign Prostatic Hyperplasia. [file 1472-6963-7-158-S1.doc]

*Knowledge questionnaire on Benign Prostatic Hypertrophy (BPH – correct answers are underlined)*

**1. Analysing the available RCT on α1-blockers in BPH:**

1. Different studies demonstrate that hypotension is less frequent with tamsulosin than with alfuzosin
2. Drugs more selective for α1 receptors are better tolerated than less selective ones
3. Among α1-blockers, tamsulosin has the best tolerability profile
4. No α1-blocker has demonstrated better tolerability than the others
5. Among α1-blockers, alfuzosin has the best tolerability profile

**2. To avoid one more case of prostate surgery in patients with BPH, it is necessary to treat with finasteride:**

1. At least 5 patients for several years
2. At least 90 patients for less than one year
3. At least 100 patients for several years
4. At least 20 patients for several years
5. At least 5 patients for less than one year

**3. Approximately, every 100 patients with BPH, a long-lasting treatment with finasteride**

1. Can avoid surgery in about 20-25 patients
2. Can avoid surgery in about 4-5 patients
3. Can affect the sexual sphere negatively in about 20-25 patients
4. Can avoid urinary retention in about 10-15 patients
5. Can avoid surgery in about 50-60 patients

**4. Dutasteride:**

1. Has fewer side effects compared to finasteride
2. Does not reduce surgery in patients with BPH
3. Reduces BPH symptoms more than finasteride
4. Is more cost-effective than finasteride
5. None of the above

**5. In BPH patients, a combination therapy with finasteride plus an alpha blocker:**

1. Reduces surgery
2. Adds on side effects of both drugs
3. Reduce symptoms in a way patients can perceive
4. a + b + c
5. None of the above

*Knowledge questionnaire on primary prevention of osteoporotic fractures (correct answers are underlined)*

**1. The decision to prescribe bisphosphonates or sr ranelate or raloxifene should not be affected by which of these conditions?**

1. Age > 65
2. Menopause occurring before 45 years of age
3. Previous fracture
4. Anticipated use of oral corticosteroids (> 5 mg prednisone or equivalents) for more than 3 months
5. Collagenopathy

**2. Considering the available studies, bisphosphonates are generally more effective**

1. In the elderly
2. In people who already suffered from osteoporotic fractures (secondary prevention)
3. In high risk patients
4. a + b + c
5. None of the above

**3. To avoid a first vertebral fracture in a post-menopausal woman it is necessary to treat for several years about:**

1. 10-15 women with alendronate
2. 50-60 women with alendronate
3. 10-15 women with risedronate
4. 50-60 women with risedronate
5. 50-60 women with ibandronate

**4. Approximately, every 100 women who have never suffered from osteoporotic fractures, a long-lasting treatment can avoid vertebral fractures**

1. With risedronate, in 5-10 women
2. With alendronate, in 5-10 women
3. With risedronate, in 1-2 women
4. With ibandronate, in 1-2 women
5. With alendronate, in 1-2 women

**5. Intramuscolar bisphosphonates**

1. Are highly absorbed
2. Can easily cause necrosis in the site of injection
3. Allow more predictable results comparing to those given through the oral route
4. Are not supported by evidence in terms of fracture reduction
5. a + b

*Attitudinal questionnaires*

*(pre-meeting; post-meeting for the discussed topic only)* **In women older than 70 with femoral T – score between – 1,5 and - 3 and without additional risk factors, you would rather consider a treatment with**

1. alendronate 70 mg/ week
2. risedronate 35 mg/ week
3. ibandronate 150 mg/ week
4. strontium ranelate 2 g / day
5. vitamin d 400 UI / day + calcium
6. vitamin d 800 UI / day + calcium
7. no drug (but would increase physical activity)

*(pre-meeting; post-meeting only for the discussed topic)* **In patients with less than 60 years with moderate BPH symptoms and prostate volume of about 30 ml, you would rather consider a treatment with**

1. Finasteride
2. Dutasteride
3. Tamsulosin
4. Alfuzosin
5. Terazosin
6. Doxazosin
7. A combined therapy (5-ARI inhibitor + alpha blocker)
8. No drug and watchful waiting (considering the possibility of surgery in the future)

*(post-meeting)* **At first look, in your opinion, the information reported provide:**

|  |  | very much | | | quite much | | | | | not so much | | | | not at all | | | |
| --- | --- | --- | --- | --- | --- | --- | --- | --- | --- | --- | --- | --- | --- | --- | --- | --- | --- |
| a good overview of the available knowledge |  |  |  |  |  |  |  |  |  | |  |  |  |  |  |  |  |
|  |  |  |  |  |  |  |  |  |  |  |  |
|  |  |  |  |  |  |  |  |  |  |  |  |
| an unbiased interpretation of this knowledge |  |  |  |  |  |  |  |  |  | |  |  |  |  |  |  |  |
|  |  |  |  |  |  |  |  |  |  |  |  |
|  |  |  |  |  |  |  |  |  |  |  |  |
| useful knowledge for your clinical practice |  |  |  |  |  |  |  |  |  | |  |  |  |  |  |  |  |
|  |  |  |  |  |  |  |  |  |  |  |  |
|  |  |  |  |  |  |  |  |  |  |  |  |
